# Kaposi Sarcoma-Associated Immune Reconstitution Inflammatory Syndrome (KS-IRIS) in Advanced HIV: Challenges in Managing Visceral Disease With Pericardial Involvement

**DOI:** 10.1093/ofid/ofag363

**Published:** 2026-06-18

**Authors:** Natalie A Sturd, Nicole L Kusnik, Katerina Roma, Alexander Hewlett, Sara H Bares, Jennifer M Davis

**Affiliations:** Department of Medicine, University of Nebraska Medical Center, Omaha, Nebraska, USA; Division of Infectious Diseases, University of Nebraska Medical Center, Omaha, Nebraska, USA; Division of Gastroenterology and Hepatology, University of Nebraska Medical Center, Omaha, Nebraska, USA; Division of Gastroenterology and Hepatology, University of Nebraska Medical Center, Omaha, Nebraska, USA; Division of Infectious Diseases, University of Nebraska Medical Center, Omaha, Nebraska, USA; Division of Infectious Diseases, University of Nebraska Medical Center, Omaha, Nebraska, USA

**Keywords:** advanced HIV, HIV, IRIS, Kaposi sarcoma, pericardial effusion

## Abstract

Immune reconstitution inflammatory syndrome (IRIS) is a well-described complication following initiation of antiretroviral therapy (ART) in people with HIV. In this case report, we describe the complex clinical presentation of a 27-year-old ART-naïve male with advanced HIV who developed Kaposi sarcoma-associated IRIS (KS-IRIS) with visceral involvement and pericardial effusion shortly after starting ART. We review known risk factors for KS-IRIS, its clinical manifestations including rare cardiac involvement and paradoxical worsening with steroid use, as well as current evidence for chemotherapeutic and antiviral treatment strategies.

Immune reconstitution inflammatory syndrome (IRIS) is a well-described complication following antiretroviral therapy (ART) initiation in people with HIV (PWH), particularly among those with advanced disease and is associated with significant morbidity and mortality. Diagnosis is nuanced, requiring clinical judgment and exclusion of alternative etiologies. The reported incidence of IRIS varies by population and geographic region, ranging between 7% and 13% in treatment-naïve patients. Common syndromes include tuberculosis-IRIS, *Mycobacterium avium* complex-associated IRIS, CMV immune reconstitution uveitis, and cryptococcal IRIS [1–3]. IRIS associated with Kaposi sarcoma (KS) accounts for ∼6% of IRIS cases, though incidence varies by region [1, 4]. We report a case of a 27-year-old man with advanced HIV who developed paradoxical worsening of visceral KS following ART initiation, complicated by pericardial effusion with tamponade attributable to KS-IRIS.

## CASE REPORT

A 27-year-old man with recently diagnosed advanced HIV with low CD4 count not yet on ART presented to the emergency department with a 5-day history of worsening chest tightness and dyspnea. He reported fatigue, fever, dysphagia, odynophagia, and abdominal pain. Vitals were as follows: temperature 100.7°F, heart rate 141 bpm, SpO2 96% on room air at rest, and blood pressure 104/71 mmHg. Physical examination revealed oral thrush, splenomegaly, and three small, isolated violaceous lesions involving the dorsum of the left foot, hard palate, and labial frenum (Figure 1). Laboratory evaluation was notable for a hemoglobin of 7.0 g/dL (13.3–17.3 g/dL)], hyponatremia [sodium 127 mmol/L (136–145 mmol/L)] and elevated liver enzymes [aspartate aminotransferase (AST) 84 U/L (15–41 U/L), alanine aminotransferase (ALT) 68 U/L (7–52 U/L)], and alkaline phosphatase 382 U/L (32–91 U/L)]. Total bilirubin was 0.3 mg/dL (0.3–1.0 mg/dL). Laboratory studies obtained 2 weeks prior demonstrated a CD4 count of 139 cells/µL (8.7%), CD4:CD8 ratio 0.15 (reference range 0.80–3.90), and HIV-1 RNA level of 144 000 copies/mL. Computed tomography (CT) scan of the chest, abdomen, and pelvis demonstrated diffuse bilateral tree-in-bud nodularity (Figure 4*A*), diffuse lymphadenopathy, splenomegaly, and cholelithiasis with gallbladder wall thickening and pericholecystic fluid.

**Figure 1.**
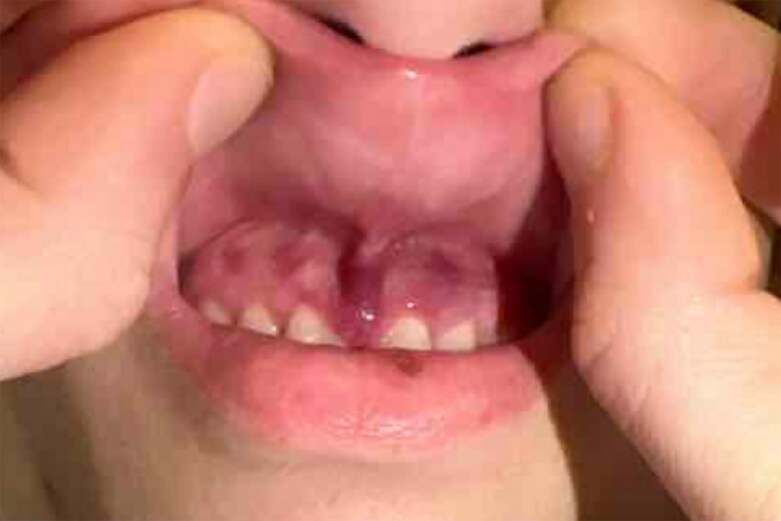
Violaceous mucosal lesions involving the labial frenulum, consistent with Kaposi sarcoma. Multiple raised purple nodules are visible along the gingival mucosa, characteristic of mucocutaneous involvement in advanced HIV infection.

After admission, he was started on empiric intravenous ceftriaxone and oral doxycycline for suspected community-acquired pneumonia plus metronidazole for possible intra-abdominal infection. Testing for other potential co-infections was notable for a (1,3)-beta-D-glucan >500 pg/mL (reference range, <80 pg/mL), though *Pneumocystis jirovecii* (PJP) PCR of sputum was not detected. The patient was treated for presumed PJP with high-dose trimethoprim-sulfamethoxazole. *Toxoplasma* IgG was positive at 11.2 (reference range ≤8.8 IU/mL). Serum *Cryptococcal* antigen was negative, and the patient had tested negative for syphilis 2 weeks prior. Given the negative serum cryptococcal antigen and lack of neurologic signs or symptoms, an LP was not performed. Following tuberculosis rule-out, defined here as two negative sputum acid fast bacilli (AFB) smears and nucleic acid amplification tests for *Mycobacterium tuberculosis* (MTB), he was started on ART with bictegravir/emtricitabine/tenofovir alafenamide on hospital day (HD) 4. Additionally, disseminated *Histoplasma capsulatum* infection was diagnosed based on positive urine and serum *Histoplasma* antigen testing. AFB blood culture obtained for disseminated *M. avium* complex (dMAC) rule-out grew *H. capsulatum*. The patient was started on intravenous liposomal amphotericin B (3 mg/kg every 24 h) for 14 days and then transitioned to oral itraconazole (200 mg 3× daily ×3 days loading dose followed by 200 mg twice daily). Biopsies of the oral lesions confirmed Kaposi sarcoma.

On HD 14, the patient developed hematemesis and melena. Esophagogastroduodenoscopy (EGD) revealed innumerable pedunculated, vascular, violaceous nodules of varying sizes throughout the stomach (Figure 2*A* and 2*B*) with biopsies confirming visceral KS, despite having minimal cutaneous disease. Oncology was consulted for systemic chemotherapy recommendations. However, the oncology team elected to defer chemotherapy given the concern for worsening disseminated histoplasmosis with further immune suppression. On HD 22, he developed worsening dyspnea, increasing oxygen requirements, and tachycardia. CT chest demonstrated a large pericardial effusion (Figure 3) and transthoracic echocardiography revealed early tamponade. Pericardiocentesis drained 700 mL of cloudy orange fluid. Empiric steroids were initiated for presumed myopericarditis. Pericardial fluid cytology demonstrated many leukocytes and numerous discohesive, mildly atypical round to spindle-shaped cells with immunohistochemical staining consistent with KS. There were no large anaplastic cells or findings otherwise concerning for a B-cell lymphoma, though flow cytometry was not performed. Given the paradoxical worsening combined with a rapid decline in HIV-1 RNA (232 copies/mL at 3 weeks of ART; 2.8 log_10_ decrease), there was concern for Kaposi sarcoma IRIS (KS-IRIS).

**Figure 2.**
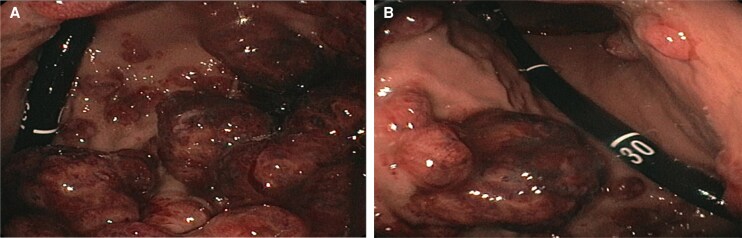
*A* and *B*: Medium to large (10–30 mm) pedunculated polyps with violaceous nodular lesions throughout the gastric body, visualized on EGD.

**Figure 3.**
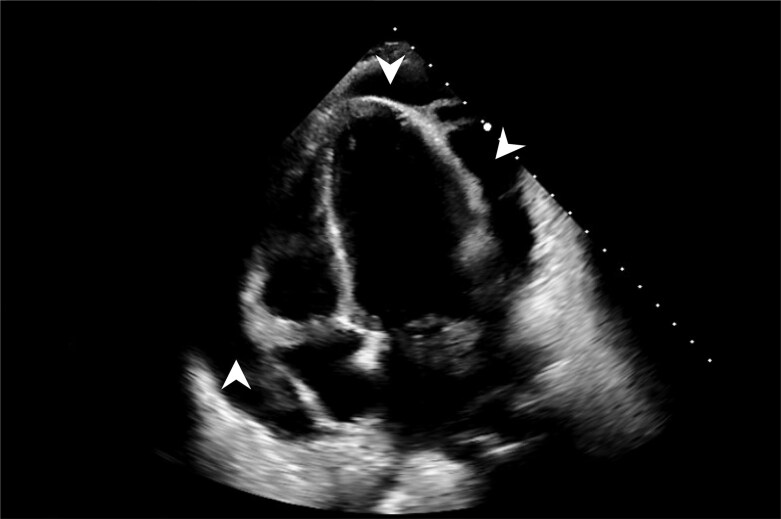
Transthoracic echocardiogram (apical four-chamber view) demonstrating a large circumferential pericardial effusion. The effusion surrounds the cardiac chambers and was later confirmed to be secondary to Kaposi sarcoma on pericardial fluid cytology.

On HD 27, the patient started liposomal doxorubicin chemotherapy (20 mg/m^2^ every 3 weeks) for KS. He gradually improved, and he was discharged to an acute rehabilitation facility after ∼40 days. Discharge medications included bictegravir/emtricitabine/tenofovir alafenamide, voriconazole 300 mg twice daily (changed from itraconazole due to drug–drug interactions and subtherapeutic levels), trimethoprim-sulfamethoxazole 400–80 mg daily for PJP prophylaxis, and a prednisone taper (40 mg daily followed by 11-week taper, reducing the dose every 4–5 days).

Ten days after discharge, he was readmitted with worsening shortness of breath, increased oxygen requirements (from room air to 2 L/min), and tachycardia. Initial workup demonstrated leukocytosis (24.5 × 10^3^/µL) and CT chest showed an interval increase in bilateral patchy opacities (Figure 4*B* and 4*C*). Worsening respiratory status prompted intubation and bronchoscopy with bronchoalveolar lavage (BAL) which was notable for an elevated *Histoplasma* antigen (>25.0 ng/uL) and human herpesvirus 8 (HHV8) PCR (4 160 000 copies/mL, 7.6 log copy/mL). BAL fluid cultures grew extended spectrum beta-lactamase (ESBL) *Escherichia coli* (>100 000 CFU/mL). Abdominal imaging demonstrated infarcts to the kidneys and spleen and occlusion of the celiac artery. The patient's clinical status rapidly deteriorated, leading to lactic acidosis and progressive shock requiring maximum pressors. The patient was transitioned to comfort measures and passed shortly thereafter. An autopsy was not performed, so the cause of death was never definitively identified.

**Figure 4.**
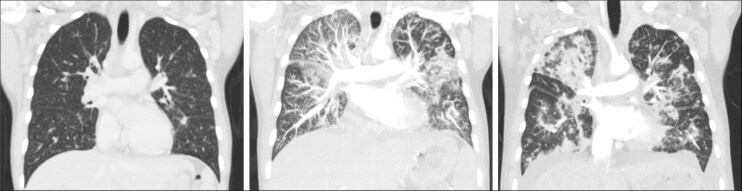
*A*, CT Chest on first admission, *B*, CT Chest while in rehabilitation facility following discharge, *C*, CT Chest on readmission. 4B and 4C show extensive bilateral patchy consolidative lung opacities.

## DISCUSSION

### KS-IRIS: Presentation, Epidemiology, and Risk Factors

IRIS presents as two distinct temporal patterns. The most common presentation is paradoxical IRIS, where existing opportunistic infections or neoplasms significantly worsen following initiation of ART, whereas with unmasking IRIS, these conditions are only discovered following ART initiation [5]. Proposed criteria for KS-IRIS [6] are outlined in Table 1, though should be considered with any worsening KS disease following ART initiation. KS-IRIS most commonly occurs within days to 6 months following ART initiation, with a median onset of 8–12 weeks [5, 7–9]. However, more recent data suggest KS-IRIS may develop much sooner, sometimes within 1 week after initiating current first-line ART. KS-IRIS clinical presentation depends on the site of involvement: new or worsening mucocutaneous lesions, new or worsening gastrointestinal (GI) bleeding or obstruction with GI involvement, or respiratory failure in those with pulmonary KS [4, 5, 10–13]. Cardiac manifestations are much rarer and are addressed later in this discussion.

**Table 1.** Proposed Criteria for Diagnosis of KS-IRIS, as Described in the Kaposi Sarcoma NCCN Guidelines Version 2.2026. Diagnosis of KS-IRIS Requires at Least One Major and One Minor Criteria

Proposed Criteria For Diagnosis Of KS-IRIS
Major Criteria
New onset or enlargement of KS lesion and subsequent regression
Painful lesions
Minor Criteria
Decrease in plasma HIV RNA by >1 log10 copies/mL
Increased blood CD4 T-cell count after ART

Risk factors shared among all forms of IRIS include low baseline CD4 counts and rapid restoration of CD4 counts [14, 15]. A prospective cohort study of 69 individuals identified several independent predictors of KS-IRIS, including (1) HIV-1 RNA >100 000 copies/mL, (2) hematocrit less than 30%, and (3) detectable plasma HHV-8 DNA [16]. Our patient had both an HIV-1 RNA of 140 000 copies/mL and a hematocrit of 23.1% when he initiated ART. However, a serum HHV-8 qPCR was not obtained, since diagnosis was made via tissue biopsies. Currently, there are no standardized US guidelines addressing HHV-8 serum PCR testing aside from suspected KICS (KSVH-Associated Inflammatory Cytokine Syndrome) [17–19].

KS disease burden should also be considered when assessing risk of KS-IRIS. T1 staging of KS is a risk factor for KS-IRIS, independent of initial CD4 cell counts [9]. While it is intuitive that greater KS disease burden increases the risk of IRIS, the extent of visceral disease may not be evident at initial presentation, as with this case. Notably, up to 40% of visceral KS cases may occur without a sentinel mucocutaneous lesion [11]. In short, the absence of mucocutaneous lesions should not exclude the diagnosis of visceral KS in this population, and clinicians should be wary of more extensive disease.

### Prevention of KS-IRIS: Evidence for Chemotherapy and Antivirals

Early ART initiation is the most effective measure for preventing KS [6, 19], as low baseline CD4 cell counts and unsuppressed viremia are strong risk factors for its development. In patients with limited mucocutaneous disease, management of KS typically involves ART alone. Systemic chemotherapy plus ART is recommended in patients with higher disease burden, including extensive cutaneous disease, KS-related edema, visceral disease, and KS-IRIS [20]. Liposomal anthracyclines (eg, liposomal doxorubicin) are the preferential first-line systemic therapy [21], though paclitaxel is an alternative if the patient has cardiac comorbidities or to treat recurrence after treatment with liposomal doxorubicin [6]. Other complications of HHV-8 disease, including multicentric Castleman's disease and primary effusion lymphoma, would also be treated differently based on the diagnosis. However, it is unclear how systemic chemotherapy impacts incidence or outcomes of KS-IRIS in treatment-naïve patients beginning ART. In one prospective cohort study of 129 patients with stage T1 KS, investigators compared liposomal anthracyclines plus ART to ART alone and found a reduction in relative risk for development of KS-IRIS in those treated with ART plus chemotherapy (RR 0.49; 95% CI 0.16 to 1.55) [22]. However, a meta-analysis of 792 PWH with severe KS failed to show a reduction in KS-IRIS in patients treated with ART plus chemotherapy [23].

While no antivirals are definitively shown to treat HHV-8, a recent study evaluated whether treatment with valganciclovir prior to starting ART decreased the incidence and mortality of KS-IRIS in 40 individuals with disseminated KS [24]. Mortality attributable to KS-IRIS was not statistically different between treatment groups [24]. Thus, the role of anti-HHV-8 antivirals as prophylaxis against KS-IRIS in disseminated KS requires further investigation.

### KS-IRIS, Cardiac Involvement, and Steroid Therapy

Another key topic of this patient's case was appropriate management of his pericardial effusion and presumed myopericarditis with cytology consistent with KS. While pericardial effusion is relatively common in PWH (incidence between 11% and 21%) [25–29], the etiology is usually infectious or neoplastic. KS pericardial involvement is rarely reported [30], and KS-associated pericardial effusion accounts for only ∼5% of pericardial effusions in this population [31–33].

This case illustrates a central dilemma of treating inflammatory conditions (eg, myopericarditis) when the patient has concurrent KS or KS-IRIS. While other varieties of IRIS may be managed with NSAIDs and corticosteroids [4, 15], steroids are contraindicated in the management of KS [5] as they have been shown to paradoxically worsen KS-IRIS and increase the risk of KS-associated mortality [34–36]. One case-controlled study of 145 individuals with HIV-associated KS on ART investigated glucocorticoid exposure as a risk factor for KS-IRIS and mortality [35]. Glucocorticoid use was more frequent in individuals with KS-IRIS than in those without (54.9 vs 36.47%, *P* = .047), and glucocorticoids were a risk factor for mortality (adjusted odds ratio = 4.719, 95% confidence interval=1.383–16.103, *P* = .0132). Mechanistically, glucocorticoids can directly stimulate KS cell proliferation via the KS-associated herpesvirus lytic cycle and promote angiogenesis [35, 37]. This patient was started on corticosteroids for presumed myopericarditis before pericardial fluid cytology demonstrated pathology consistent with KS, which likely increased the risk of worsening KS-IRIS manifestations. Currently, corticosteroid use in KS-IRIS is guided by expert consensus and individualized risk–benefit assessment. This case highlights the need for prospective studies or consensus guidelines addressing management of inflammatory complications of KS-IRIS, particularly when corticosteroid-responsive comorbidities are concomitant.

## CONCLUSIONS AND FINAL THOUGHTS

This case highlights critical teaching points regarding the recognition and management of KS-IRIS. First, the impact of early HIV diagnosis on the prevention of immune reconstitution syndromes cannot be overstated. Second, a complete physical examination including a thorough skin and oropharyngeal exam to identify mucocutaneous KS lesions is essential for all PWH, particularly those with advanced disease. Even in patients without identifiable lesions, clinicians should maintain a low threshold of suspicion for visceral involvement in patients with advanced disease and risk factors for IRIS. Vigilant monitoring for early indications of all reconstitution inflammatory syndromes should be a primary goal in care of these patients.

This case also highlights the complexities surrounding glucocorticoid use in KS-IRIS. Though corticosteroids are contraindicated in KS-IRIS due to the increased risk of morbidity and mortality, there may be cases involving comorbidities requiring steroid therapy, during which patients should receive the shortest course possible. Given the limited evidence guiding management of KS-IRIS, early involvement of oncology is essential to guide decisions regarding systemic chemotherapy. In summary, hospitalized patients with advanced HIV benefit from multidisciplinary care and require consistent re-evaluation to assess for emerging complications, including IRIS. Although KS-IRIS is uncommon, awareness of shared risk factors and early manifestations may facilitate timely diagnosis and improve outcomes in this high-risk population.
